# An automated system for the measurement of hydrogen peroxide in industrial applications

**DOI:** 10.1155/S1463924698000236

**Published:** 1998

**Authors:** Philippe Westbroek, Edward Temmerman, Paul Kiekens, Filip Govaert

**Affiliations:** 1 Department of Analytical Chemistry University of Gent Krijgslaan 281 S12 Gent B 9000 Belgium; 2 Department of Textile Technology University of Gent Technologiepark 9 Zwijnaarde B 9052 Belgium; 3 Department of Analytical Chemistry Krijgslaan 281 S12 Gent B 9000 Belgium

## Abstract

An automated sensor system for the continuous and in-line measurement of hydrogen peroxide in industrial applications is described. The hydrogen peroxide concentration can be measured over the entire pH range, over a wide concentration range of hydrogen peroxide (10^-3^ 70 g/l), from 0 to 70^°^C, and with high precision and accuracy (errors less than 1% ). The system consists of a bypass in which the necessary electrodes are positioned and electronically controlled. The sensor is very selective for hydrogen peroxide, easy to instal, and it is stable for at least two months after calibration. The calibration can be done in the process solution during a running process.


Journal of Automatic Chemistry, Vol. 20, No. 6 (November–December 1998) pp. 185–188

### An automated system for the measurement of hydrogen peroxide in industrial applications

**Philippe Westbroek, Edward Temmerman**
*Department of Analytical Chemistry, University of Gent, Krijgslaan 281 S12, B 9000, Gent, Belgium*
**Paul Kiekens**
*Department of Textile Technology, University of Gent, Technologiepark 9, B 9052 Zwijnaarde, Belgium*
**and Filip Govaert**
*Department of Analytical Chemistry, Krijgslaan 281 S12, B 9000 Gent, Belgium*

*An automated sensor system for the continuous and in-line measurement of hydrogen peroxide in industrial applications is described. The hydrogen peroxide concentration can be measured over the entire pH range, over a wide concentration range of hydrogen peroxide ($10^{-3}$–70 g/l), from 0 to 70°C, and with high precision and accuracy (errors less than 1%). The system consists of a bypass in which the necessary electrodes are positioned and electronically controlled. The sensor is very selective for hydrogen peroxide, easy to instal, and it is stable for at least two months after calibration. The calibration can be done in the process solution during a running process.*

### Introduction

Despite the weaker oxidizing properties [1] of hydrogen peroxide when compared with chlorite [2] and hypochlorite [2], hydrogen peroxide has been the most widely used oxidizing agent for the last 20 years. This is because it has environmental and ecological advantages [3]. Its reaction products are oxygen and water [3] rather than the cumbersome chlorine compounds formed in reactions of chlorite and hypochlorite [4].

It is often necessary to measure concentrations of hydrogen peroxide, monitoring its level (for example in environmental analysis) and to control its concentration at a present value (for example bleaching processes) to obtain the best possible quality of the bleached products. A continuous analysis method is frequently called for.

Over the past 30 years numerous methods have been developed to measure hydrogen peroxide concentration, but none of them has been able to improve on the classical titration of hydrogen peroxide by hand with potassium permanganate [5]. Trials with automatic titrators [6] gave satisfying results, but the cost is relatively high and it is a discontinuous determination. Other techniques, like conductometric, potentiometric [7] and calorimetric [8] methods, could be used as continuous measurements, but their precision and accuracy is too low. Colorimetric [8] methods measure the hydrogen peroxide concentration continuously (with an FIA system) with high precision and accuracy, but the cost of the spectrophotometer means that industrial implementation on a large scale has never been done.

This paper describes a sensor system that can measure and control the hydrogen peroxide concentration in industrial processes, making use of the amperometric principle [9] with a three-electrode potentiostatic set-up [9] equipped with a combined glass electrode and PT100 probe to measure pH and temperature.

### Description of the system

### *Working principle*

Hydrogen peroxide can be oxidized and in voltammetry gives rise to a classical anodic wave. In sufficiently alkaline solutions, and thanks to a specific pretreatment procedure [10] carried out at the surface of the glassy carbon electrode, a special oxidation reaction of hydrogen peroxide can be obtained [10]. This produces a wave situated at less positive potentials than the classical wave. This special reaction was used in the amperometric three-electrode set-up used in the investigation described here. A constant potential difference of +0.5 V [11] was applied between the working (i.e. hydrogen peroxide sensitive electrode made of glassy carbon) and the reference electrode ($Ag/AgCl/Cl^-$). The advantage of this special reaction over the common oxidation reaction is that hydrogen peroxide concentrations up to 70 g/l can be measured (for the common oxidation reaction the upper limit is 0.1 g/l [10]) and the electrode surface is stable for at least two months after pretreatment. A disadvantage of this reaction is the need for an alkaline environment. The reaction only occurs in a pH range from 10.5 to 14. This problem can be overcome with a FIA system.

The hydrogen peroxide concentration can be calculated from the measured current after compensation for pH and temperature differences between the measurement of an unknown concentration (m) and the calibration measurement (cal) because the current is dependent on pH and temperature. This calculation is done with the aid of equation (1), which shows that the relationship between hydrogen peroxide concentration and sensor output current is rather complicated and has to be solved by iterations:

$$c_{H_2O_{2,m}} = \left[ c_{H_2O_{2,cal}}^{[1.5-(0.5kc^y_{H_2O_{2,cal}})]} \frac{I_m}{I_{cal}} \frac{c_{OH^-_{cal}}^{[(1.5kc^y_{H_2O_{2,cal}})-0.5]}}{c_{OH^-_m}^{[(1.5kc^y_{H_2O_{2,m}})-0.5]}} \times e^{0.03049(T_{cal}-T_m)} \right]^{1/[1.5-(0.5kc^y_{H_2O_{2,m}})]} \quad (1)$$

0142-0453/98 $12·00 © 1998 Taylor & Francis Ltd where $c_{H_2O_2}$ is the concentration of hydrogen peroxide, $c_{OH^-}$ is the hydroxide concentration, $I_m$ and $I_{cal}$ are the current output signals for measurement and calibration, $T_m$ and $T_{cal}$ are temperature for measurement and calibration, $k = 0.6567$ and $y = -0.07643$.

The resulting concentration can be used as parameter for the control of the hydrogen peroxide concentration during the process.

*Bypass configuration*

A bypass is used for implementation of the sensor system into the intended process. Two types of bypass are used: one for processes carried out in the pH range from 10.5 to 14, [12], and the other is modified with an FIA system for use in applications carried out at pH values below 10.5. It is the FIA system that is described in this paper.

Because the specific oxidation reaction of hydrogen peroxide on which the sensor is based only occurs if pH is higher than 10.5, the pH of a process liquid that is lower than 10.5 has to be increased. A flow injection system is used to do this (figure 1). A constant flow of process liquid is mixed with an identical flow of a hydroxide solution. Dependent on the pH of the process liquid, the hydroxide solution is more or less diluted. This causes an increase in the pH of the mixed solution to at least 12, at which the value oxidation reaction takes place while the hydrogen peroxide concentration is diluted by a factor of two. In the detection area (or analysis area) the amperometric current, pH and temperature are measured, allowing the hydrogen peroxide concentration to be calculated using equation (1). The parameters needed to obtain this concentration were measured in the bypass, so the result has to be multiplied by two to give the original hydrogen peroxide concentration in the process tank. This can be incorporated directly in the iterative calculation by inserting the number 2 in front of the brackets on the right-hand side of equation (1).

*Electronic device*

The electronic device includes:

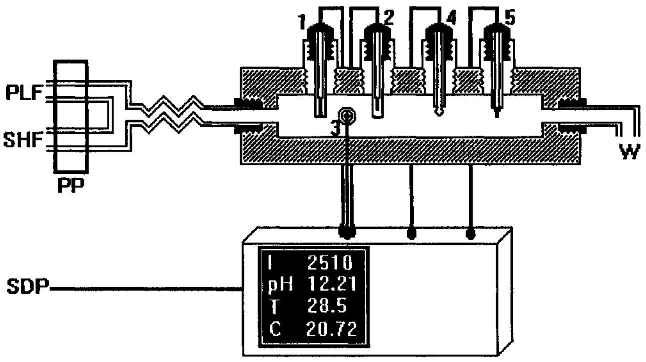

*Figure 1. Flow injection analysis system for the continous measurement of hydrogen peroxide. PLF = process liquid flow, SHF = sodium hydroxide flow, W = waste and SDP = signal for dosing pumps; 1 = working electrode, 2 = reference electrode, 3 = counter electrode, 4 = combined pH electrode and 5 = PT100.*

- Calibration procedures for the pH electrode and the hydrogen peroxide sensitive electrode system (three electrode set-up).
- Conversion of the experimental output signals obtained from the electrodes and amplification of these signals.
- Software to calculate the hydrogen peroxide concentration from the converted parameters.
- Control units to control the hydrogen peroxide concentration and/or the pH of the process.

### Experimental

The working electrode and the counter electrode were made by the authors. The working glassy carbon disk electrode was produced by embedding a glassy carbon rod (Le Carbon Lorraine, France, type V20) in epoxy resin (figure 2). The counter electrode was made in a similar way. The reference electrode and PT100 were commercial products from Jumo (Germany). The combined pH-electrode was obtained from Schott (Germany). These electrodes were chosen because of their good stability, relatively long lifetime and sufficiently rugged construction. The electrodes were held in a home-made bypass tube made from polypropylene and polyvinylchloride, both obtained from Stockvis Plastics (Belgium). The electronic device in the sensor system was developed and built by Inverto (Belgium). It offers a number of user options. For the preliminary experiments (such as determination of the interrelationships between amperometric output current signal, pH, temperature and hydrogen peroxide concentration), use was made of a PGSTAT10 potentiostat from Eco chemie (Netherlands), controlled by a software program (GPES4.3) from the same company. To control the apparatus and to process the obtained experimental data, a PC486DX2 from Eknadata (Belgium) was used.

### Results and discussion

In order to evaluate the dependence of the amperometric sensor output on pH, temperature signal and hydrogen peroxide concentration, voltammetric experiments were conducted in which the values of the variable parameters were varied within a wide range. As can be seen from figure 3, the relationship between current signal and

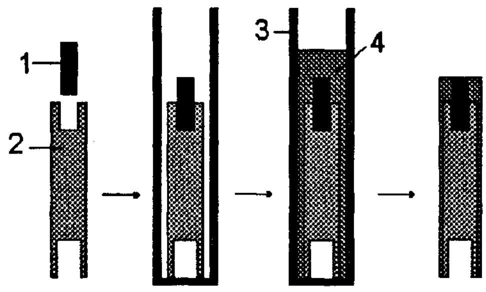

*Figure 2. Scheme of the construction of the glassy carbon electrode. 1 = glassy carbon rod, 2 = copper holder, 3 = PVC holder and 4 = epoxy resin.*

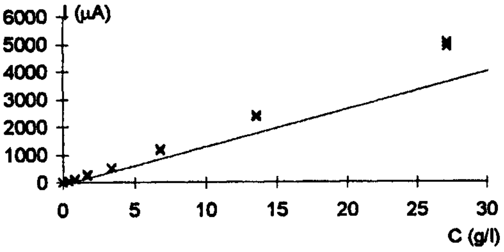

*Figure 3. Relationship between sensor output amperometric current and hydrogen peroxide concentration for the oxidation of hydrogen peroxide at the surface of a glassy carbon electrode. $E = +0.50V$ vs $Ag|AgCl|Cl$; $pH = 12.24$ and $T = 25.0\,°C$.*

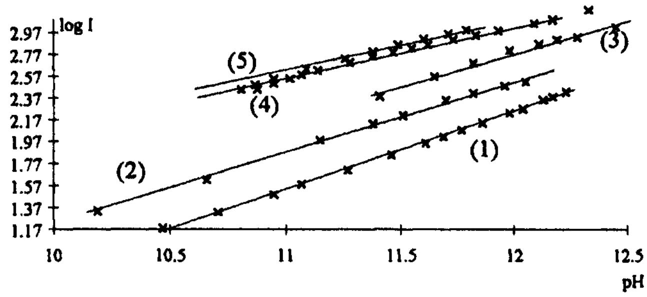

*Figure 4. Relationship between logarithm of amperometric current for the oxidation of hydrogen peroxide at the surface of a glassy carbon electrode and pH for different hydrogen peroxide concentrations. $E = +0.50V$ vs $Ag|AgCl|Cl^-$; $T = 25.6\,°C$. Concentrations of hydrogen peroxide are (1) 2 g/l, (2) 4 g/l, (3) 8 g/l, (4) 15 g/l and (5) 20 g/l.*

hydrogen peroxide concentration is not linear, but the slope (i.e. reaction order of hydrogen peroxide concentration) increases with increasing hydrogen peroxide concentration. The reaction order of the hydroxide concentration was found to decrease with increasing hydrogen peroxide concentration (figure 4). the relationship between current signal and temperature is exponential but is independent of the hydrogen peroxide concentration. Equation (1) is a detailed analysis of the relationships obtained.

The sensor system is directly applicable in processes where the pH is higher than 10.5 and has been successfully installed in several applications. The advantage of this direct application is the possibility of using large bypass tubes, which strongly minimizes the risk of blockage by waste, fibres, etc. Furthermore, no dilution effects are present and the bypass liquid can be pumped back to the process tank because there is no pH difference or any other contamination (no reagent is used).

This system has already been implemented in industry to measure the hydrogen peroxide concentration in washing machines where the pH is less than 9. For processes operating at pH values lower than 10.5 a sodium hydroxide solution of 0.5 g/l is used to increase the pH in a bypass flow. This configuration generates a waste solution. Its amount is minimized by reducing the size of the bypass tubes, which in turn increases the risk of blocking by solid particles. The latter problem could be solved by using self cleaning filters. The experimental data (see

*Table 1. Comparison of hydrogen peroxide concentration determined in process tank ($pH = 9$) by means of permanganate titration with concentration determined with sensor in FIA bypass of tank.*

| *c(g/l) using equation (1)* | *c(g/l) using titration* |
|---|---|
| 10.06 | 10.02 |
| 8.45 | 8.45 |
| 6.19 | 6.21 |
| 4.02 | 4.01 |
| 1.99 | 2.01 |
| 1.20 | 1.19 |
| 0.66 | 0.67 |
| 0.35 | 0.35 |
| 0.20 | 0.19 |

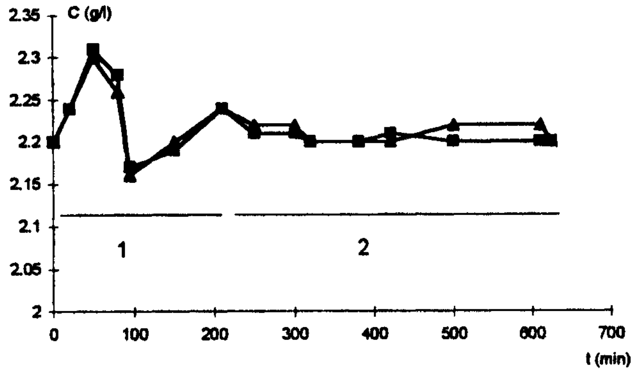

*Figure 5. Hydrogen peroxide concentration as a function of time during a running washing process. pH of the process is about 9, pH in the bypass is around 12; (1) measuring of hydrogen peroxide concentration, hydrogen peroxide is added manually; (2) measuring and control of the hydrogen peroxide concentration; concentration obtained by titration (■) and by using equation (1) (▲).*

table 1) show that the sensor system measures the hydrogen peroxide concentration with deviations that never exceed 1%. The concentration of hydrogen peroxide is verified by titration of a sample taken at the same moment as the registration of the electrode signals.

As can be seen from figure 5 the sensor system not only can measure (part 1) the hydrogen peroxide concentration but also can control it (part 2) to a preset value (2.2 g/l) with high precision and accuracy. Table 1 shows the result of varying the hydrogen peroxide concentration.

The sensor system for hydrogen in general is highly selective. This is due to the fact that it is based on the oxidation of hydrogen peroxide. If an oxidizable substance enters the process solution it would be oxidized by hydrogen peroxide to a higher oxidation state due to the oxidizing properties of the reagent. This also means that this substance already oxidized by hydrogen peroxide, cannot be further oxidized at the electrode surface at the applied potential.

### Conclusion

The sensor system described can measure hydrogen peroxide concentrations over a wide concentration and temperature range, with high precision and accuracy. when the pH is higher than 10.5 it can be used for process solutions. With an FIA attachment, it is suitable for the whole pH range. The sensor system reacts immediately when the concentration changes, it is stable without recalibration for at least two months after pretreatment and calibration, it is very selective for hydrogen peroxide, easy to instal, easy to handle and is relatively low cost.

### References

1. Hoare, J. P., 1985, Oxygen, in A. J. Bard, R. Parsons and J. Jordan (eds), *Standard Potentials in Aqueous Solution* (New York: John Wiley and Sons).
2. Rosch, G., 1988, *Textil Praxis International*, **4**, 384.
3. Weck, M., 1991, *Textil Praxis International*, **2**, 144.
4. Grunwald, W., 1961, *Zeitschrift Gesellschaft Textilindustrie*, **63**, 285.
5. Schumb, W. C., Satterfield, C. N. and Wentworth, R. L., 1955, *Hydrogen Peroxide* (New York: Reinhold).
6. Laube, K. and Zollinger, H., 1965, *Melliand Textilberichte*, **46**, 727.
7. Eisele, J. and Hafenrichter, S., 1954, *Melliand Textilberichte*, **35**, 756.
8. Jola, M., 1980, *Melliand Textilberichte*, **61**, 931.
9. Kissinger, P. I. and Heinemann, W. R., *Laboratory Techniques in Electronanalytical Chemistry* (New York: Marcel Dekker).
10. Westbroek, P., Temmerman, E. and Kiekens, P., 1998, *Analytical Communications*, **35**, 21.
11. Westbroek, P., Van Haute, R. and Temmerman, E., 1996, *Fresenius Journal of Chemistry*, **354**, 405.
12. Westbroek, P., Temmerman, E and Kiekens, P., 1988, *Melliand Textilberichte*, **79**, 62.
13. Hartmann, W., 1997, *International Textile Bulletin*, **2**, 1.

